# Supplementary material for: Measurement of nanoscale three-dimensional diffusion in the interior of living cells by STED-FCS
Source: Nat Commun. 2017 Jul 6;8:65. doi: 10.1038/s41467-017-00117-2 (PMC5500520; doi:10.1038/s41467-017-00117-2)
Supplement: Supplementary file 1 — Supplementary Information [file 41467_2017_117_MOESM1_ESM.pdf]

File Name: Supplementary Information
Description: Supplementary Figures and Supplementary Note

File Name: Peer Review File
Description:

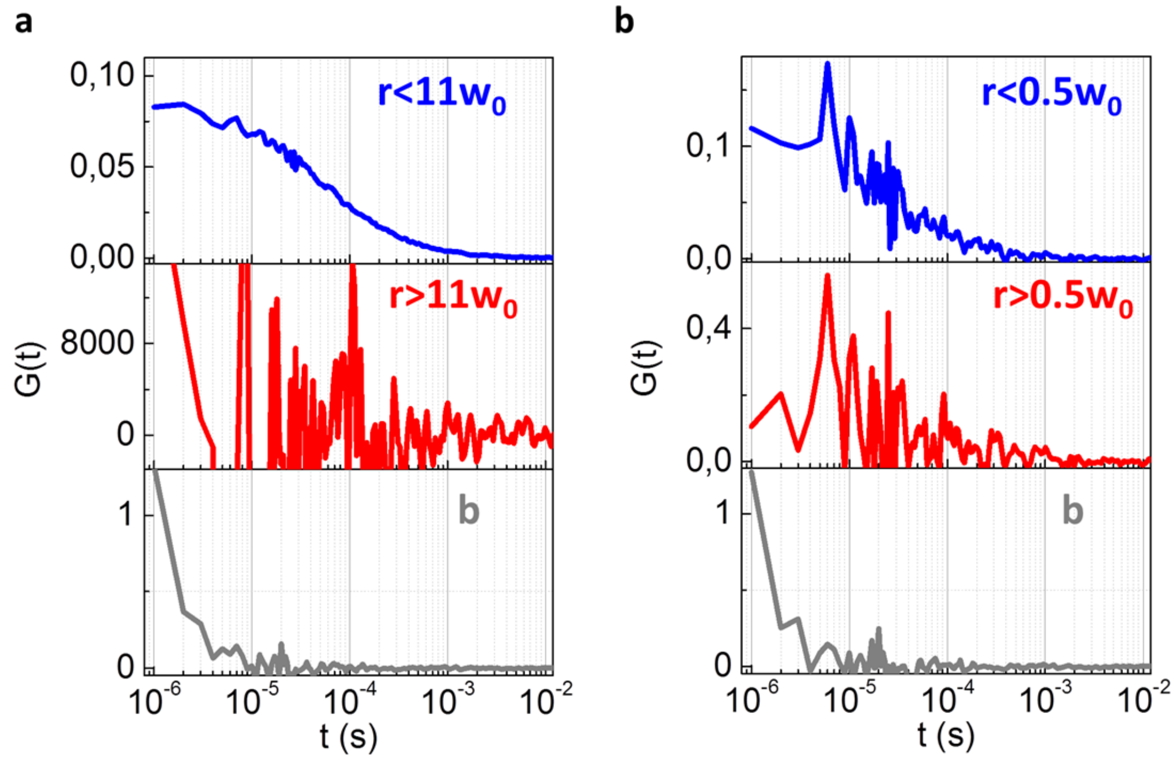

Supplementary Fig.1. Complete set of ACFs extracted by FLCS analysis. (**a,b**) ACFs obtained by FLCS analysis of a measurement of EGFP in PBS at a STED power of 50 mW. The ACFs correspond to the three temporal decay patterns defined in Eq.(5-7). The analysis is shown for r_1=11w_0 (**a**) and r_1=0.5w_0 (**b**).

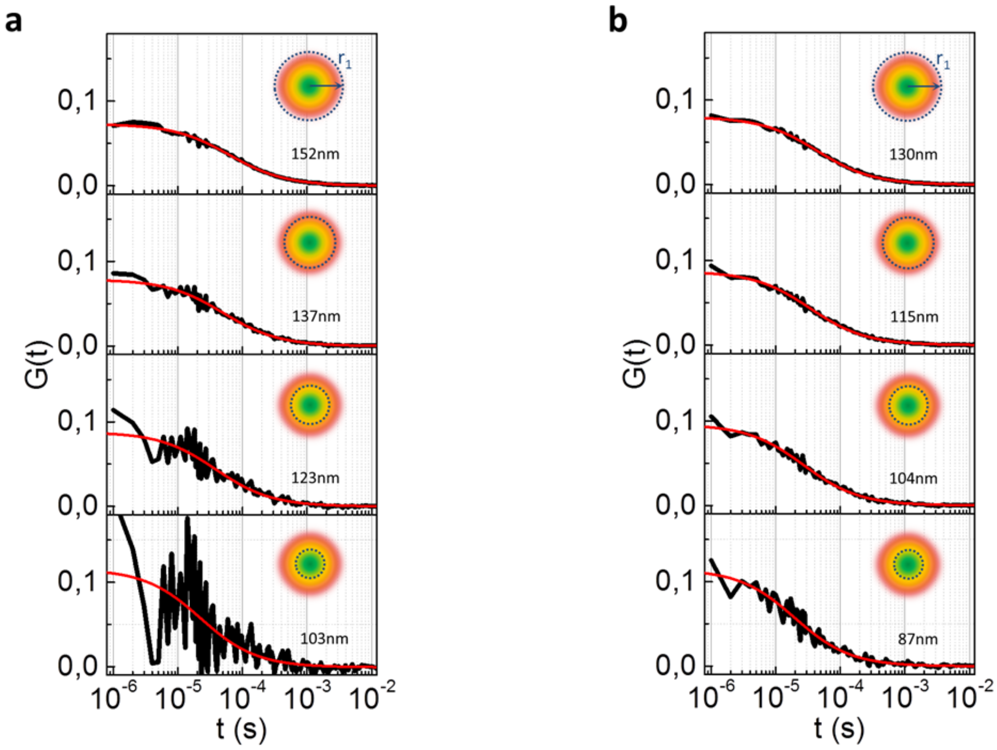

Supplementary Fig.2. SPLIT ACFs of EGFP in solution at different STED powers. (**a,b**) Filtered ACFs of EGFP in PBS obtained at a STED power of 20 mW (**a**) and 80 mW (**b**) for different values of the parameter r_1 (r_1=11w_0, 2w_0, w_0, 0.5w_0 respectively, from top to bottom). Each of the ACFs is obtained from a FLCS-based separation into three components and is associated to the calculated decay corresponding to the radial region $r<r_1$. Solid lines are a fit of the data to Eq.(15) and the numbers indicate the recovered lateral size w of the effective observation volume.

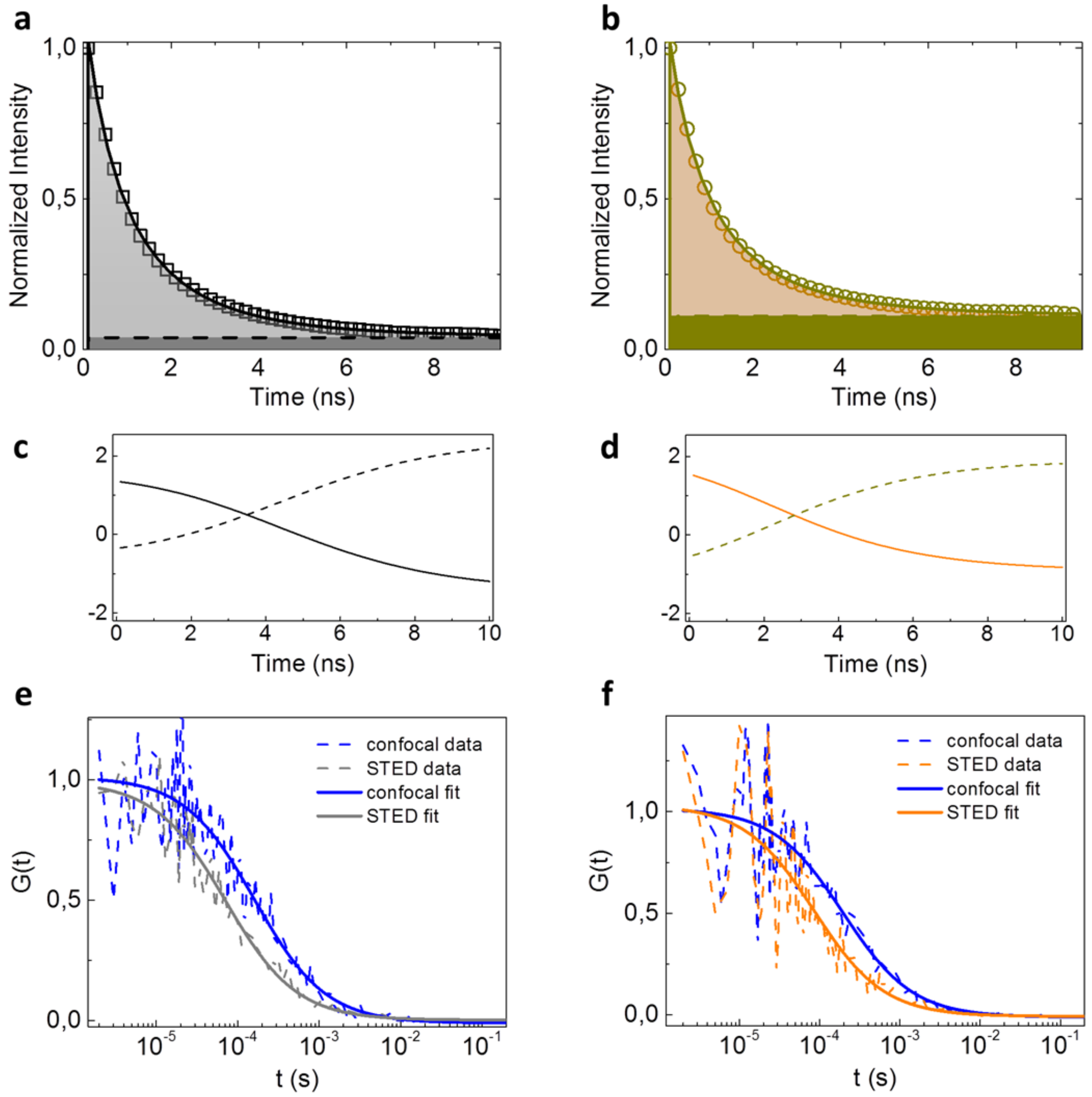

Supplementary Fig.3. Removal of uncorrelated background by FLCS. (**a-f**) Comparison between measurements of a solution of Oregon-green-488 secondary antibody in PBS at two different levels of uncorrelated background. The measurements have been performed at the same STED power (P_{STED}=120 mW) and at an excitation power of P_{exc}=4.5µW (**a**,**c**,**e**) and P_{exc}=1.5µW (**b**,**d**,**f**), respectively. (**a**,**b**) Average STED decays, background level *b*=0.039 and *b*=0.11, respectively. The shaded areas highlight which is the relative contribution of uncorrelated background photons with respect to the modulated fluorescence photons, integrated over the period *T*. (**c**,**d**) FLCS filters corresponding to the STED decay and the uncorrelated background. (**e**,**f**) The normalized STED ACFs obtained after removal of background are compared with the corresponding normalized confocal ACFs. The same decrease in transit time (t_D^{STED}/t_D^{conf}=0.46±0.05 and 0.45±0.05 for P_{exc}=4.5µW and 1.5µW respectively) in (**e**) and in (**f**) indicates that STED-induced uncorrelated background is properly filtered out by the analysis.

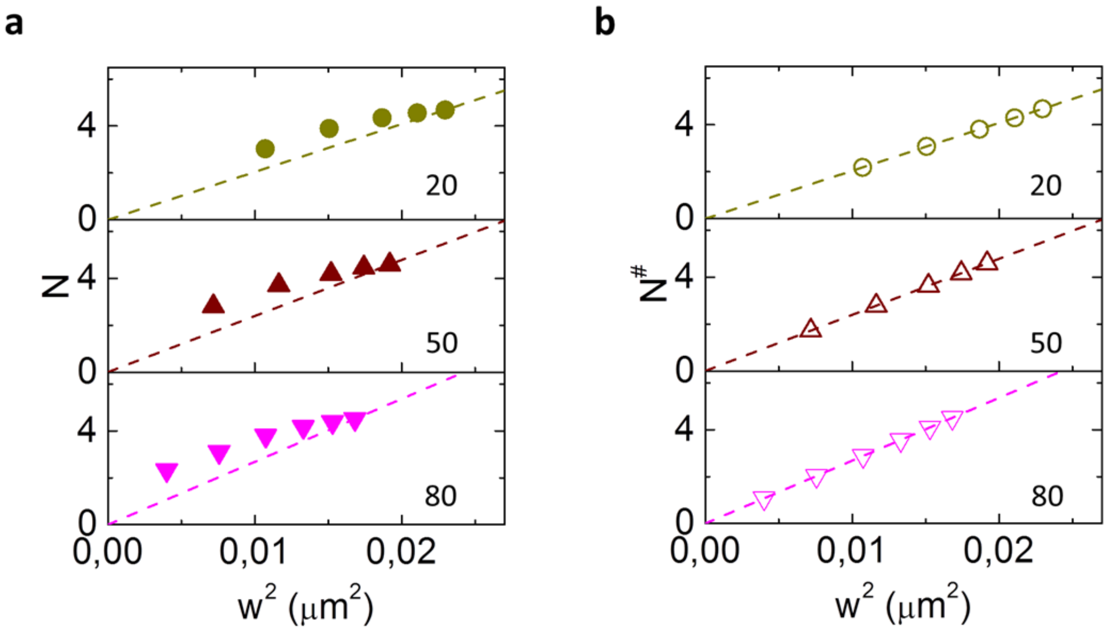

Supplementary Fig.4. Number of particle for EGFP in solution at different STED powers. (**a**) Non-corrected number of particles obtained from SPLIT-FLCS of EGFP in solution as a function of the lateral size of the observation volume. The dashed lines indicate a linear scaling and highlight the non-linear scaling of the non-corrected number of particles. (**b**) Number of particles after correction. Numbers indicate STED beam power in mW (measured at the back aperture of the objective lens).

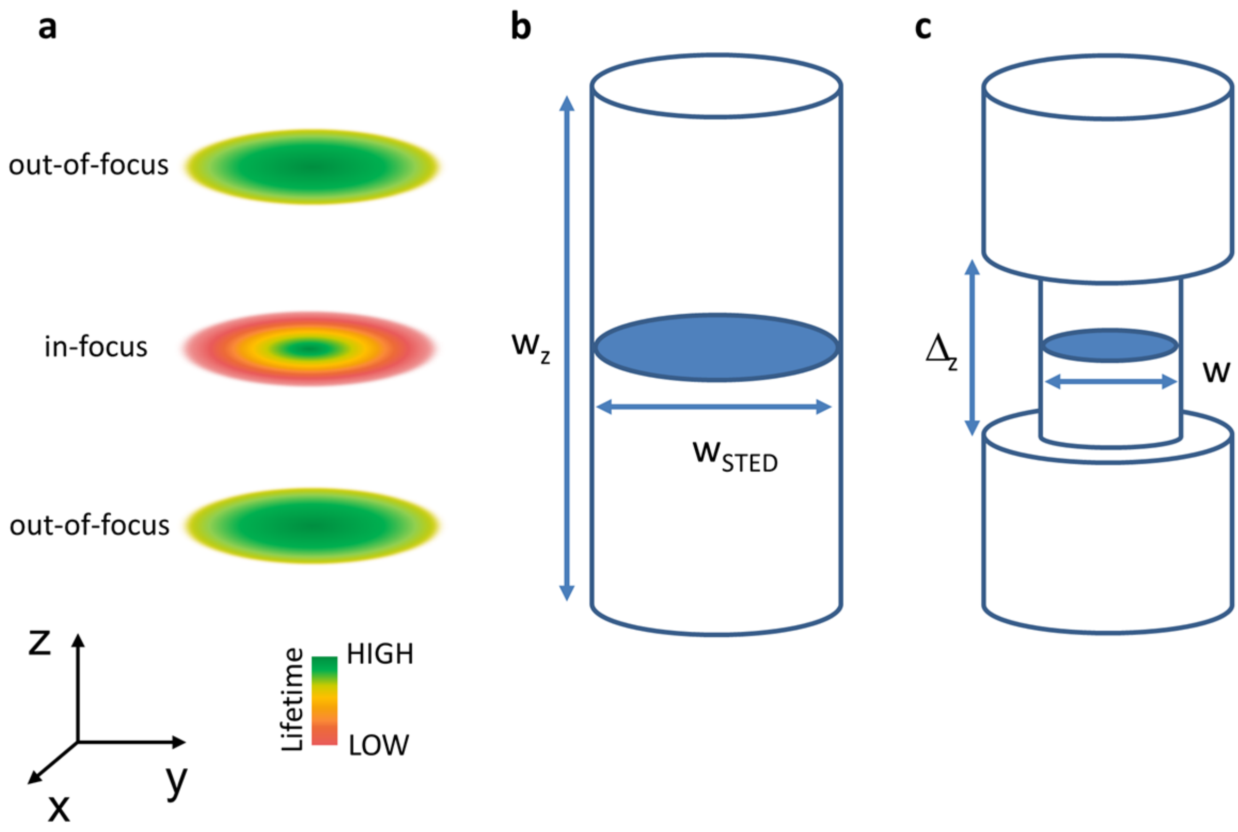

Supplementary Fig.5. Simplified model for volume shape factor. (**a**) Schematic of the distribution of lifetime at axial position near the focal plane and far from the focal plane (**b**,**c**) Sketches of the 3D observation volumes in STED (r_1>>w_0) (**b**) and SPLIT (**c**). It is assumed that the STED is effective (i.e. induces a significant gradient of lifetimes) only in a region of depth $\Delta_z < w_z$ near the focal plane. Then the two volumes are easily calculated and an approximated formula for the shape factor can be derived.

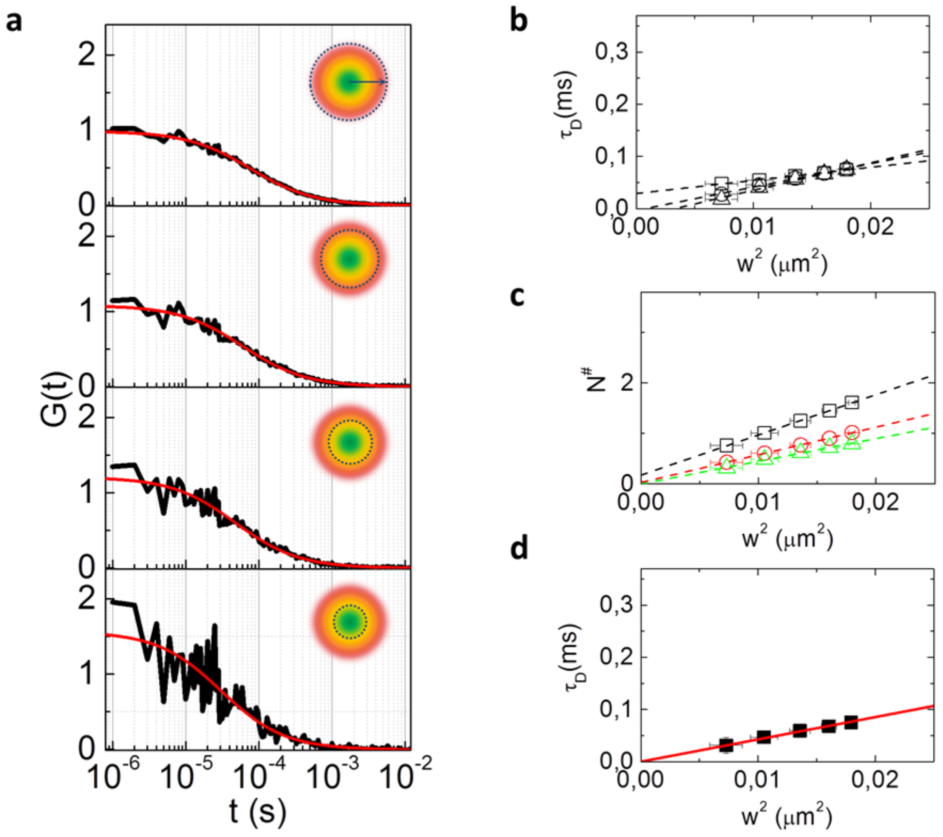

Supplementary Fig.6. SPLIT-FLCS of EGFP in a viscous solution. (**a**) Representative filtered ACFs of EGFP in a glycerol-water mixture at a STED power of 50 mW for different values of the parameter r_1. Solid lines are a fit of the data to Eq.(15). (**b**) Transit time t_D as a function of the square of the lateral size of the effective observation volume w^2, for three measurements. (**c**) Corrected value of the number of molecules $N^{\#}$ versus w^2, for the three measurements. The dashed lines are linear fits to the data. (**d**) Diffusion law obtained as the average of three measurements. The solid line is fit to Eq.(14) (D=58 $\mu m^2\ s^{-1}$).

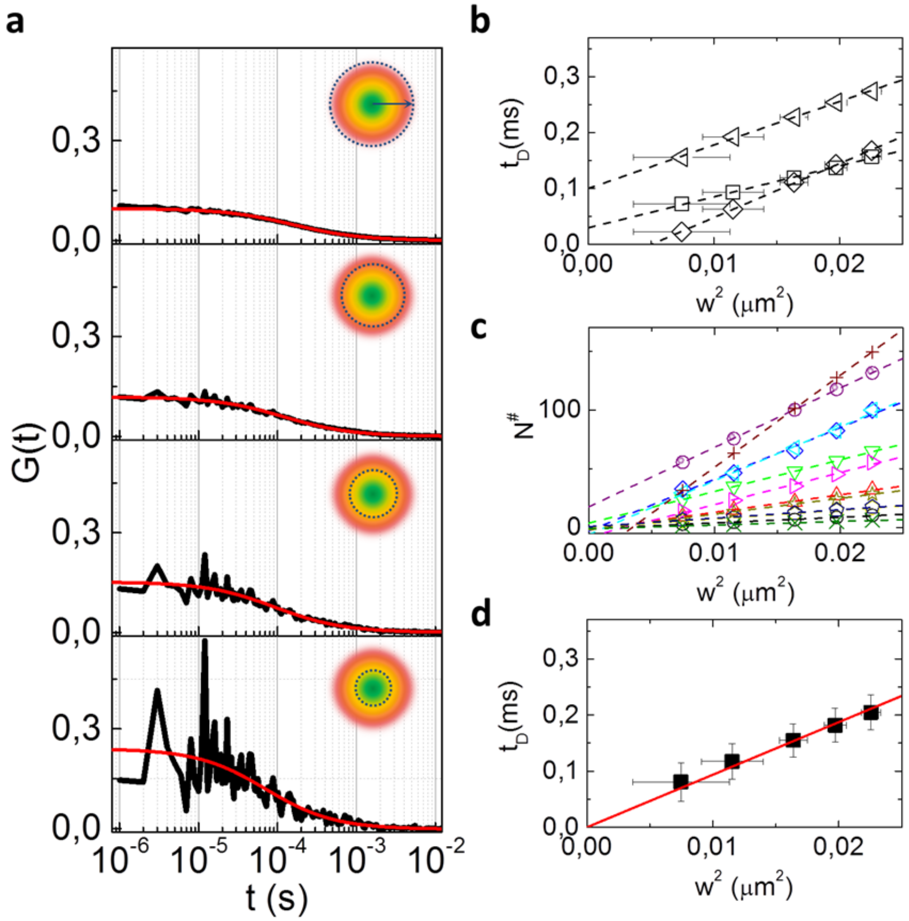

Supplementary Fig.7. SPLIT-FLCS of EGFP in the cytoplasm of HeLa cells at lower STED power. (**a**) Representative filtered ACFs of EGFP in the cytoplasm of a HeLa cell at a STED power of 20 mW for different values of the parameter r_1. Solid lines are a fit of the data to Eq.(15). (**b**) Transit time t_D as a function of the square of the lateral size of the effective observation volume w^2, for three different cells. (**c**) Corrected value of the number of molecules $N^\#$ versus w^2, for all the measured cells. The dashed lines are linear fits to the data. The slope varies from cell to cell according to differences in EGFP expression level. (**d**) Diffusion law obtained as the average of single-point measurements in the cytoplasm of n=12 different HeLa cells. The solid line is fit to Eq.(14) (D=26 μm^2 s^{-1}).

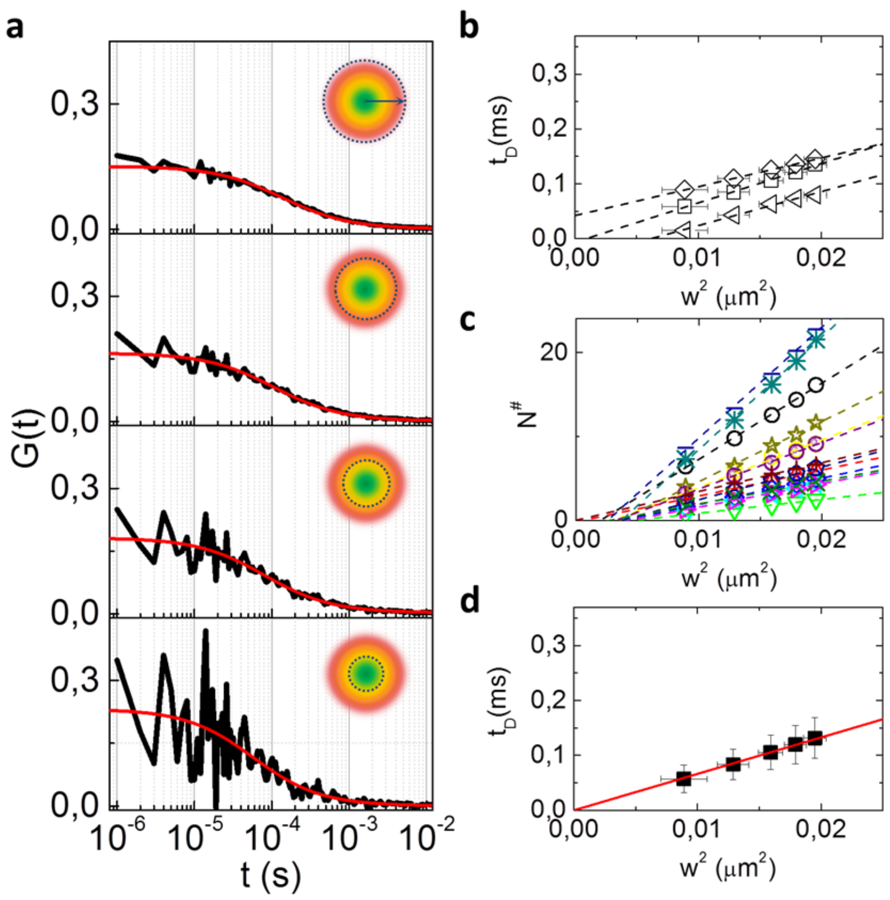

Supplementary Fig.8. SPLIT-FLCS of EGFP in the cytoplasm of CHO cells. (**a**) Representative filtered ACFs of EGFP in the cytoplasm of a CHO cell at a STED power of 50 mW for different values of the parameter r_1. Solid lines are a fit of the data to Eq.(15). (**b**) Transit time t_D as a function of the square of the lateral size of the effective observation volume w^2, for three different cells. The dashed lines are linear fits. (**c**) Corrected value of the number of molecules $N^\#$ versus w^2, for all the measured cells. The dashed lines are linear fits to the data. The slope varies from cell to cell according to differences in EGFP expression level. (**d**) Diffusion law obtained as the average of single-point measurements in the cytoplasm of n=14 different CHO cells. The solid line is fit to Eq.(14) (D=38 $\mu m^2\ s^{-1}$).

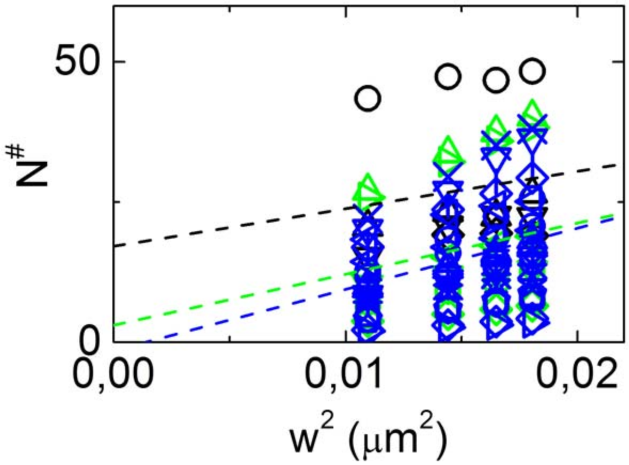

Supplementary Fig.9. Corrected value of number for tubulin-EGFP. Corrected value of the number of molecules $N^{\#}$ versus w^2, for same single-point measurements on tubulin-EGFP shown in Fig.4 in the main text. The data points have been grouped and colored as in Fig.4c. The dashed lines are linear fits of the average of each group. The group of slow diffusion modes shows a positive intercept. This is probably due to a larger immobile fraction causing a decrease in the value of the G(0) amplitude.

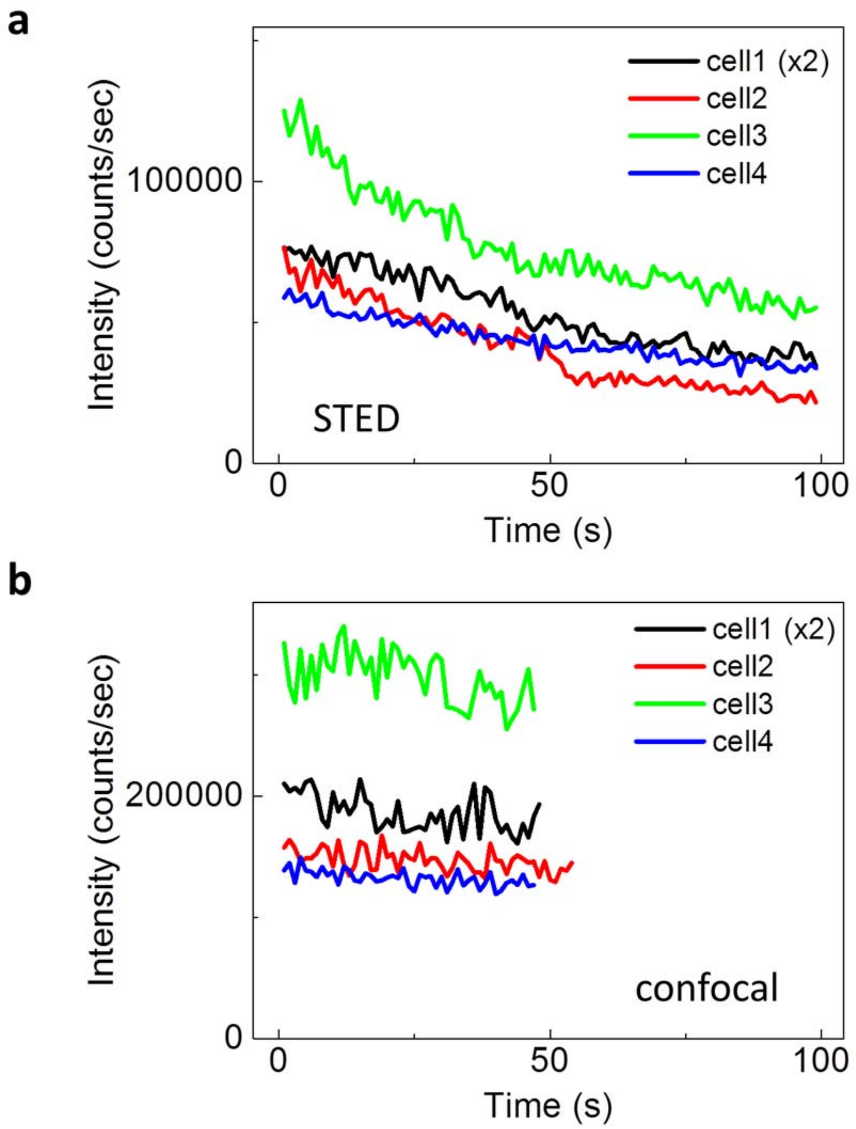

Supplementary Fig.10. Photobleaching during SPLIT-FLCS of EGFP in live cells. (**a**,**b**) Representative intensity traces recorded during single-point measurements in the cytoplasm of HeLa cells at a STED power of 50 mW (**a**) and 0 mW (**b**), respectively. Some curves have been rescaled for better visualization.

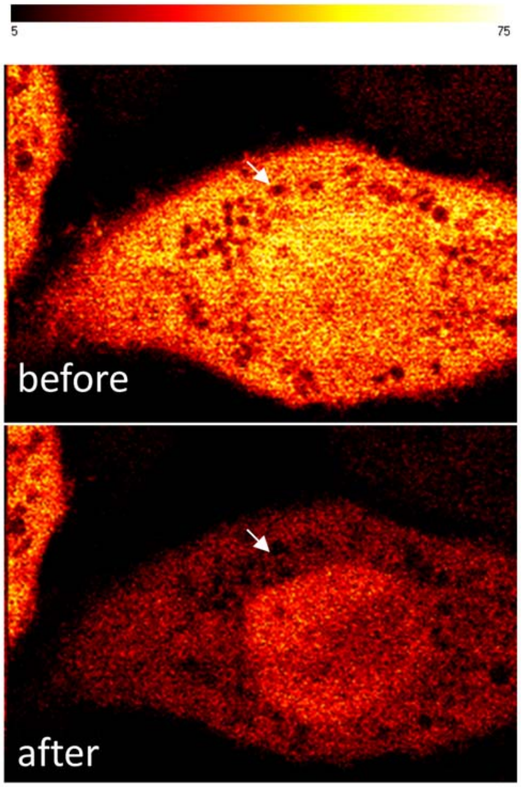

Supplementary Fig.11. Transient concentration gradient induced by photobleaching. Confocal images acquired right before and right after a 100-s long acquisition in the cytoplasm of CHO cells (in the point indicated by the arrow) at a STED power of 50 mW.

Supplementary Note 1- Derivation of the number correction factor

We assume that the STED is effective only in a region $\Delta_z < w_z$ near the focal plane (Supplementary Fig.5). Thus a significant reduction of the lateral size of the effective observation volume V is obtained only within this region but not in the out-of-focus planes. According to the simple scheme depicted in Supplementary Fig.5, the volume V can be expressed as:

$$V = V_{STED} - \Delta V = k_{vol}^{STED} w_{STED}^2 w_z - k_{vol}^{STED}\left(w_{STED}^2 - w^2\right)\Delta_z \qquad \text{(S1)}$$

Where $V_{STED} = k_{vol}^{STED} w_{STED}^2 w_z$ and $\Delta V = k_{vol}^{STED}\left(w_{STED}^2 - w^2\right)\Delta_z$.

According to the definition of s:

$$s = \frac{V}{V_{STED}}\frac{w_{STED}^2}{w^2} \qquad \text{(S2)}$$

We can write:

$$s = \frac{k_{vol}^{STED} w_{STED}^2 w_z - k_{vol}^{STED}\left(w_{STED}^2 - w^2\right)\Delta_z}{k_{vol}^{STED} w_{STED}^2 w_z}\frac{w_{STED}^2}{w^2} = \frac{w_{STED}^2 w_z - \left(w_{STED}^2 - w^2\right)\Delta_z}{w^2 w_z} \qquad \text{(S3)}$$

Reordering we obtain:

$$s = \frac{w_{STED}^2}{w^2} - \left(\frac{w_{STED}^2}{w^2} - 1\right)\left(\Delta_z / w_z\right) \qquad \text{(S4)}$$

Finally the shape factor s can be expressed as:

$$s \approx \frac{w_{STED}^2}{w^2}\left(1 - \xi_z\right) + \xi_z \qquad \text{(S5)}$$

Where we have defined $\xi_z=\Delta_z/w_z$.
